# Comprehensive analysis of grazing intensity impacts on different types of grassland in Xinjiang, China: a meta-analysis

**DOI:** 10.3389/fpls.2025.1633065

**Published:** 2025-08-29

**Authors:** Yu Zheng, Huifang Yao, Kairui Chao, Xiuzhi Ma, Yanan Ma

**Affiliations:** Forestry College, Inner Mongolia Agricultural University, Hohhot, China

**Keywords:** Xinjiang grassland, grazing intensity, soil properties, community characteristics, meta-analysis

## Abstract

Grazing is the primary use of grassland in Xinjiang and plays a key role in the grassland ecosystem. Grazing intensity has a profound impact on the healthy development of grassland. To date, we lack a comprehensive understanding of the overall response of the main types of grassland in Xinjiang to different intensities of grazing. Based on 73 peer-reviewed studies, we conducted a meta-analysis of the response of soil properties and community vegetation characteristics to grazing intensity in five main types of grassland in Xinjiang. The results showed the following: (1) Grazing reduced the soil total carbon (TC) and total potassium (TK) of grassland soil in Xinjiang and increased the content of available phosphorus (AP) in soil. Moderate grazing reduced the soil TC and increased the total nitrogen (TN). Heavy grazing significantly reduced soil organic carbon (SOC) and density (*P* < 0.05). (2) The SOC of temperate meadow steppe decreased significantly under moderate and heavy grazing, and the three grazing intensities significantly reduced the biomass carbon storage of living plants and litter carbon storage (*P* < 0.05). (3) Soil pH and AP in temperate steppe increased significantly under light grazing, and soil bulk density (BD) and total phosphorus (TP) increased significantly under heavy grazing (*P* < 0.05). (4) The soil water content (SWC) and pH of temperate desert steppe decreased significantly under moderate grazing. Heavy grazing increased soil BD. Light grazing increased pH and available nitrogen (AN), and decreased soil TN, TP, and TK (*P* < 0.05). (5) The soil organic matter of mountain meadow increased significantly under light and moderate grazing. Light grazing reduced aboveground and underground standing crop and root-shoot ratio, and increased carbon storage (*P* < 0.05). (6) Under heavy grazing, SWC and total biomass in alpine meadow decreased significantly, whereas pH, AN, and AP increased significantly. Soil BD decreased significantly under light and moderate grazing (*P* < 0.05). The structural equation model showed that the increase in grazing intensity would reduce the vegetation coverage of the community and thus would reduce species diversity. The increase in grazing intensity also had a significant negative effect on aboveground biomass and soil quality.

## 1 Introduction

As an important part of the terrestrial biosphere, grassland accounts for 40% of its area (Scurlock and Hall, 1998). It not only provides a habitat for the diversity of animals and plants (Conant et al., 2001) but also contributes to the livelihood of more than 1 billion people around the world (Boval and Dixon, 2012). A stable grassland ecosystem provides an important material basis for the development of animal husbandry and the maintenance of terrestrial ecosystem balance (Buisson et al., 2022). The stability of the grassland ecosystem is affected by many factors, including vegetation, soil properties, climatic conditions, and human activities (Liu et al., 2019). Research has shown that overgrazing causes grassland ecosystem imbalance. Because of the bottleneck associated with the imbalance of grassland ecosystems in different regions and different types of grassland, the formulation of grazing measures according to local conditions can provide a scientific basis for the sustainable and stable development of grassland ecosystems (Conant et al., 2017). The grassland area in Xinjiang ranks third in China. This area is an important part of China’s grassland ecosystem and plays a key role in biodiversity conservation and the animal husbandry economy. Influenced by its distinct natural environment and landform, Xinjiang grassland has formed a variety of complex grassland types (Zhao and Jing, 2022). Because of global warming, rapid population growth, and a substantial increase in the number of livestock, Xinjiang grassland has experienced varying degrees of degradation and faces enormous challenges (Xu et al., 2022). In recent years, many studies have examined grassland degradation in Xinjiang, including grassland degradation and soil carbon composition and stability (Xu et al., 2022), characteristics and storage capacity of degraded grassland (Liu et al., 2013), sustainable development direction of degraded grassland (Kemp et al., 2020) and improvement measures (Liu et al., 2025).

Grazing is the primary way grassland is utilized, and its impact on grassland ecological diversity has always been the focus of ecological research (Herrero and Thornton, 2013). Litter in grasslands is closely related to vegetation and soil. Livestock feeding and other behaviors affect litter decomposition, change the soil’s physical and chemical properties and nutrient content, and then affect vegetation characteristics and community composition structure (Liu et al., 2008). Different grazing intensities have different effects on the physical and chemical properties of soil. The trampling of sheep and goats in the Sahara region has led to the fragmentation of soil crusts, and the area of soil crusts has been reduced. Heavy grazing reduces the soil permeability index, and moderate grazing intensity increases soil permeability (Hiernaux et al., 1999). Kobayashi et al. (1997) found that grazing reduced soil moisture on the sunny slope but did not have a significant effect on soil moisture on the shady slope. The grazing intensity of alpine grassland in the northern foot of the Tianshan Mountains in China increased by 1, and the soil compaction increased by 1.13 times (Li et al., 1993). Because of increased grazing intensity, the excretion of livestock also increased, which created more nitrogen sources for grassland. The total nitrogen content of soil was much lower than that of heavy grazing in moderate and light grazing (Yan et al., 2014). After long-term continuous grazing, the total phosphorus in the soil increased, and the content of available phosphorus also increased (Xi et al., 2009). In the natural grassland of the agropastoral ecotone in the Songnen Plain, Wang and Sheng, (2012) found that the number of soil bacteria and fungi showed a single peak curve as grazing intensity increased, and the number of soil microorganisms was highest in moderate grazing. A study by Tan et al. (2015) in temperate meadow steppe showed that as grazing intensity increased, the soil microbial biomass and the number of bacteria decreased first and then increased. Zhang et al. (2000) showed that as grazing intensity increased, the height of the plant community decreased, the surface coverage decreased, the aboveground biomass decreased greatly, and the biomass of high-quality forage decreased rapidly. Fuhlendorf and Smeins (1999) pointed out significant differences in grassland biomass under different grazing treatments. Overgrazing not only affected the growth and biomass of various organs of the aboveground part of the herbage, but also inhibited the growth and biomass formation of the underground part of the root system, resulting in grassland degradation. The research to date on grazing intensity on grassland in Xinjiang has been concentrated primarily in Yili (Wu et al., 2024a) and the northern slope of the Tianshan Mountains (Zhang et al., 2023; Li et al., 2022). The research has focused mostly soil on properties (Wu et al., 2024a), vegetation (Sun et al., 2014), and community characteristics (Dong et al., 2016). No independent research has examined the effects of grazing intensity on soil properties and vegetation characteristics of different types of grassland in Xinjiang, thus arriving at a systematic conclusion.

Therefore, in this study, we focused on five primary grassland types in Xinjiang—temperate meadow steppe, temperate steppe, temperate desert steppe, mountain meadow, and alpine meadow—to investigate grazing impacts through meta-analysis. We synthesized peer-reviewed studies (published since 2000) examining the effects of grazing intensity on soil physicochemical properties, vegetation characteristics, and plant community structure. Our objectives were as follows: (1) to compare grazing impacts on soil and vegetation dynamics across these grassland types; (2) to assess whether increasing grazing pressure induced homogenization of soil properties and community characteristics (e.g., species richness, abundance) among types; and (3) to quantify the effects of defined grazing intensities—light grazing (three or less sheep units/ha), moderate grazing (four to six sheep units/ha), and heavy grazing (seven or more sheep units/ha)—on soil and community attributes within each type of grassland. The results of this research provide a theoretical foundation for sustainable grassland utilization and optimized conservation strategies in Xinjiang.

## 2 Materials and methods

### 2.1 Experimental area

The study area is located in the Xinjiang Autonomous Region in northwest China (34°25’–48°10’N, 73°40’–96°18’E), which is deep inland and far from the ocean. It has a temperate continental climate with a large temperature difference, sufficient sunshine (annual sunshine time is 2500–3500 h), reduced precipitation, and an annual average precipitation of about 150 mm. The precipitation varies significantly from place to place. The temperature in southern Xinjiang is higher than that in northern Xinjiang, and the precipitation in northern Xinjiang is higher than that in southern Xinjiang. The average temperature in January in Junggar Basin is –20°C; the average temperature in July in Turpan is 33°C.

### 2.2 Data collection

To construct a comprehensive database of grazing intensity effects on Xinjiang grasslands, we collected peer-reviewed publications from January 2000 to December 2024 using the Web of Science (http://apps.webofknowledge.com) and the China Knowledge Resource Integrated Database (http://www.cnki.net). The search term combinations were: “grazing or herbivory or fencing”, “Xinjiang grassland or Xinjiang grassland degradation”, and “grassland type”. Next, we screened the publications to identify appropriate studies based on the following criteria: (1) The selected article had to study the impact of grazing on grassland in Xinjiang. The study also had to provide the effects of grazing intensity, grazing mode, grassland degradation, and related variables on vegetation productivity, vegetation characteristics, plant productivity, diversity index, soil quality, carbon storage, and plant production. (2) The article had to provide information such as latitude and longitude coordinates, altitude, precipitation, test duration, and the degree of grassland degradation in the study area. (3) The article had to contain a control group (no grazing) and an experimental group (grazing), and data for the control group and the experimental group had to include sample size, mean, and standard deviation (SD) or standard error (SE). Some of the data could be directly extracted from the content and tables in the article, and some of the data in the figure needed to be extracted by using WebPlotDigitizer software. For data that provided only SE and sample size (*n*), we calculated the SD according to the following formula:


$$SD=SE\times \sqrt{n}$$

**Figure 1:**
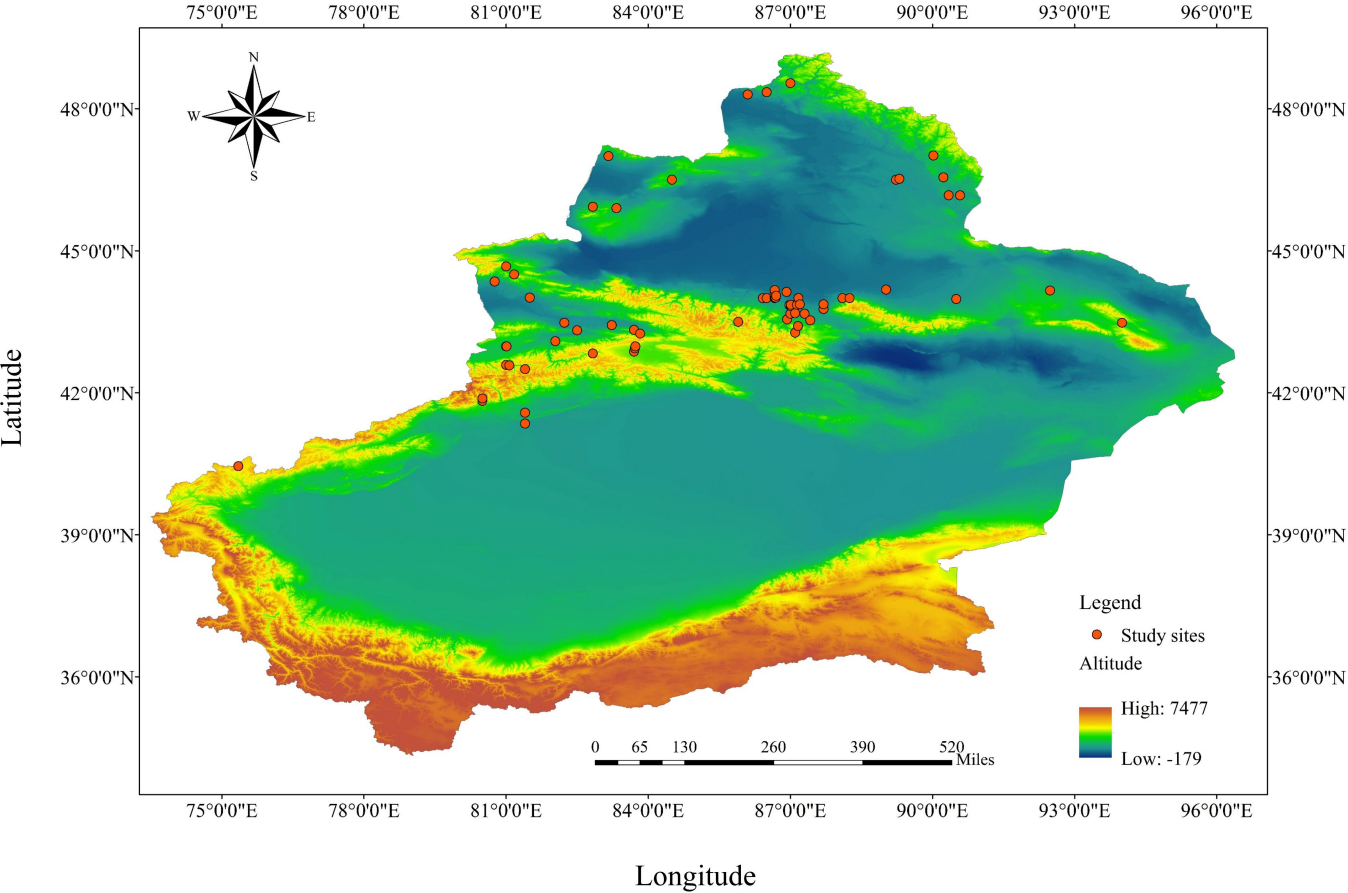
Location of study sites in the literature. The base map is made from the standard map of the Ministry of Natural Resources. The review number is GS (2023) 2767, and the map boundary is not modified.

We identified 73 journal papers that met the noted criteria (**Figure 1**). For the grassland type division and distribution map, see reference Zhang et al. (2020a). The distribution of the 73 sample plots collected was as follows: 14 temperate meadow steppes, 7 temperate steppes, 16 temperate desert steppes, 22 mountain meadows, and 14 alpine meadows. After extracting the data and removing duplicates or statistically insignificant data from each study, we created a database containing 1876 groups was created. The main indicators were as follows: bulk density (BD), soil water content (SWC), total carbon (TC), soil organic carbon (SOC), organic carbon density (OCD), soil organic matter (SOM), total organic matter (TOM), pH, total nitrogen (TN), total phosphorus (TP), total potassium (TK), available nitrogen (AN), available phosphorus (AP), available potassium (AK), carbon-to-nitrogen ratio (C:N), carbon-to-phosphorus ratio (C:P), nitrogen-to-phosphorus ratio (N:P), community vegetation height (CVH), community vegetation cover (CVC), community vegetation density (CVD), root-to-stem ratio (R:S), total biomass (TB), aboveground biomass (AGB), belowground biomass (BGB), aboveground standing crop (ASC), belowground standing crop (BSC), Shannon–Wiener index (SWI), Simpson index (SI), Pielous index (PI), species richness (SR), Patrick index (PAI), Margalef index (MI), Chao index (CI), carbon storage in live grassland biomass (CSLGB), litter carbon storage (LCS), root carbon storage (RCS), total carbon storage (TCS), and dry matter (DM). We divided grazing intensity grading standards into three levels according to Reynaud and Lanzanova (2017), as shown in **Table 1**: light grazing, moderate grazing, and heavy grazing.

**Table 1:** Grazing intensity division standard.

Grazing intensity	pasture utilization ratio (%)	Number of herbivores per hectare
Control group (non-grazing)	0	0
Light grazing	0-30	1-3
Moderate grazing	30-60	4-6
Heavy grazing	>60	>7

### 2.3 Data analyses

In this paper, we used the response ratio (*RR*) of the natural logarithm proposed by Hedges et al. (1999) to evaluate the effects of grazing intensity on soil physical and chemical properties and vegetation community characteristics of various grassland types in Xinjiang. We defined *RR* as the natural logarithm of the ratio of the mean value of the index in the test group (*X_e_*) to the mean value of the index in the control group (*X_c_*). The calculation formula is as follows:


$$RR=ln^{\left(\frac{X_{e}}{X_{c}}\right)}=ln^{\left(X_{e}\right)}-ln^{\left(X_{c}\right)}$$

The variance (*v*) of the *RR* is calculated as follows:


$$v=\frac{SD_{e}^{2}}{\left(N_{e},X_{e}^{2}\right)}+\frac{SD_{c}^{2}}{\left(N_{c},X_{c}^{2}\right)}$$

In the formula, *N_e_* and *N_c_* are the sample sizes of the experimental group and the control group, respectively, and *SD_e_* and *SD_c_* are the standard deviations of the indicators in the experimental group and the control group, respectively. The weighting coefficient (*w*) of each response ratio is the reciprocal of the variance (*v*). The calculation formula is as follows:


$$w=\frac{1}{v}=\frac{1}{SD_{e}^{2}/(N_{e},X_{e}^{2})+SD_{c}^{2}/(N_{c},X_{c}^{2})}$$

To improve the accuracy of the study, we also calculated the average weighted response ratio (*RR_++_*) of each experimental group and the control group. The higher the accuracy of the study, the greater the weight assigned. The calculation formula is as follows:


$$RR_{++}=\frac{\sum_{i=1}^{m}\sum_{j=1}^{k}w_{ij}RR_{++}}{\sum_{i=1}^{m}\sum_{j=1}^{k}w_{ij}}$$

In the formula, *w_ij_* is the weighting coefficient of *j*th variable in *i* group, *m* is the number of groups, and *k* is the number of pairs of indicators in the *i* group (one value of the indicator in the experimental group is paired with one value of the target variable in the control group). The calculation formula of the weighted standard deviation is as follows:


$$SD(RR_{++})=\sqrt{\frac{1}{\sum_{i=1}^{m}\sum_{j=1}^{k}w_{ij}}}$$

We used the 95% confidence interval (*CI*) to test whether the weighted response ratio of the indicators to the treatment in the experimental group was significant. If the 95% *CI* overlapped with 0, the response ratio of the index was not significant; conversely, the response ratio of the indicator was significant (Xu et al., 2023). When the maximum value of the 95% *CI* was less than 0, this indicated that grazing had a significant negative effect on the effect value of typical grassland types in Xinjiang. If the minimum value of the 95% *CI* was greater than 0, this indicated that grazing had a significant positive effect on the effect value of typical grassland types in Xinjiang. The calculation formula is as follows:


$$95\%CI=RR_{++}\pm 1.96SD(RR_{++})$$

We used the “Metafor” package in R 4.4.3 software to calculate the response ratio and 95% *CI*, and we used the “ggplot2” and “Forestplot” packages to draw the forest map. We selected some indices for a correlation analysis “Pearson” package. We established structural equation modeling (SEM) using the “lavaan” and “piecewiseSEM” packages to study the effects of grazing intensity on community species diversity (SWI, SI, PI and SR) and soil quality (SOC, TN, TP, TK, AN, AP, and AK.).

## 3 Results

### 3.1 Effects of grazing intensity on soil and community characteristics of grassland in Xinjiang

**Figure 2:**
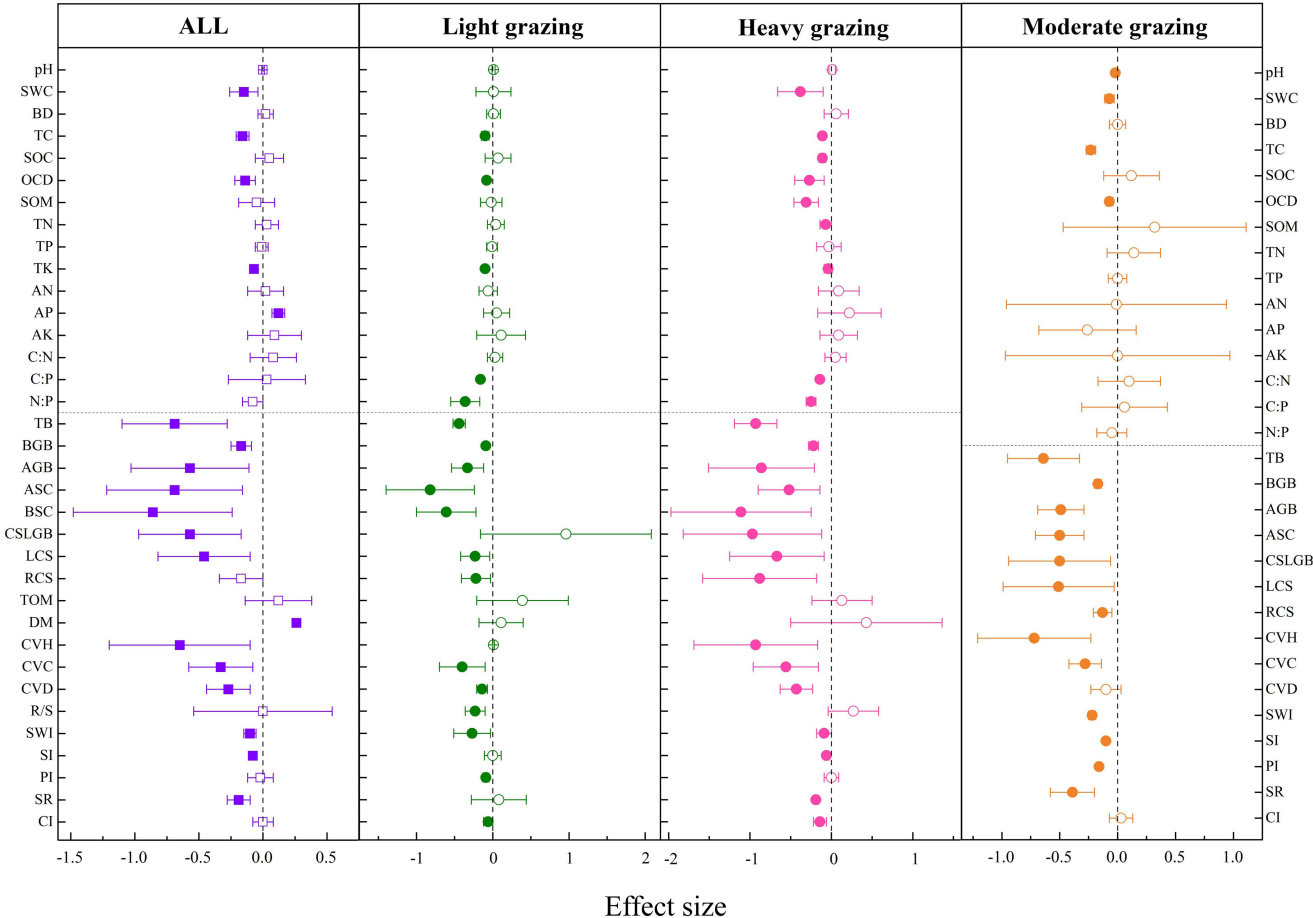
Effects of grazing and different intensities on soil and community indexes of grassland. pH, soil pH; SWC, soil water content; BD, bulk density; TC, total carbon; SOC, soil organic carbon; OCD, organic carbon density; SOM, soil organic matter; TN, total nitrogen; TP, total phosphorus; TK, total potassium; AN, available nitrogen; AP, available phosphorus; AK, available potassium; C:N, carbon-to-nitrogen ratio; C:P, carbon-to-phosphorus ratio; N:P, nitrogen-to-phosphorus ratio; TB, total biomass; BGB, belowground biomass; AGB, aboveground biomass; ASC, aboveground standing crop; BSC, belowground standing crop; TCS, total carbon storage; CSLGB, carbon storage in live grassland biomass; LCS, litter carbon storage; RCS, root carbon storage; TOM, total organic matter; DM, dry matter; CVH, community vegetation height; CVC, community vegetation coverage; CVD, community vegetation density; R/S, root-to-stem ratio; SWI, Shannon-Wiener index; SI, Simpson index; PI, Pielous index; SR, species richness; CI, Chao index. The solid representation has a significant effect, and the hollow representation has no significant effect. ”*“, “**”, and “***” are the significant codes of P (significance) < 0.05, P (significance) < 0.01, and P (significance) < 0.001 for the mean effect size, respectively.

Grazing had significant negative effects on soil total carbon (TC) (−15.93% ±0.0579 , the percentage of the response ratio, the same below), organic carbon density (OCD) (−14.4% ±0.0312 ), soil total potassium (TK) (-7.06% ±0.0284 ), community vegetation height (CVH) (−64.98% ±0.0493 ), community vegetation coverage (CVC) (−32.5% ±0.0401 ), community vegetation density (CVD) (−26.52% ±0.048 ), aboveground biomass (AGB) (−57.32% ±0.0599 ), total biomass (TB) (−69.42% ±0.1461 ), belowground biomass (BGB) (−16.91% ±0.0425 ), aboveground standing crop (ASC) (−69.11% ±0.0799 ), belowground standing crop (BSC) (85.66% ±0.1224 ), carbon storage in live grassland biomass (CSLGB) (−56.68% ±0.0849 ), litter carbon storage (LCS) (−46.29% ±0.0524 ), Shannon-Wiener index (SWI) (−10.21% ±0.0483 ), Simpson index (SI) (−8.36% ±0.0325 ) and species richness (SR) (−16.82% ±0.0477 ). We identified a significant positive effect on soil available phosphorus (AP) (11.85% ±0.0558 ) and dry matter (DM) (0.61% ±0.002 ) (**Figure 2**).

As shown in **Figure 2**, compared with the control group (no grazing), soil TC decreased significantly by 10.21% ±0.0287 and 23.25% ±0.0973 in light and moderate grazing, respectively; SOC decreased significantly by 11.42% ±0.036 in heavy grazing; OCD decreased by 6.52% ±0.0188 and 27.2% ±0.0464 in moderate and heavy grazing, respectively; soil TN increased significantly by 14.24% ±0.0438 in moderate grazing; soil TK decreased significantly by 10.01% ±0.0346 in moderate grazing; and soil AP increased significantly by 21.97% ±0.0859 in heavy grazing. C:P decreased significantly by 16.3% ±0.0823 and 14.24% ±0.0697 in light and heavy grazing, respectively, and N:P decreased significantly by 36.18% ±0.0892 and 25.26% ±0.0977 in light and heavy grazing, respectively. CVH decreased significantly by 39.87% ±0.0509 , 72.06% ±0.1169 , and 92.8% ±0.0873 in the three grazing intensities. CVC decreased significantly by 13.51% ±0.0325 , 27.82% ±0.0717 , and 56.13% ±0.0828 in the three grazing intensities. CVD was significantly reduced by 23.01% ±0.0497 and 42.96% ±0.1155 in light and heavy grazing, respectively. AGB decreased significantly by 32.92% ±0.061 , 49.25% ±0.1495 , and 85.84% ±0.1049 in the three grazing intensities. BGB was significantly reduced by 9.39% ±0.0457 , 17.08% ±0.0742 , and 22.4% ±0.0822 in the three grazing intensities. TB was significantly reduced by 64.43% ±0.1729 and 92.94% ±0.3407 in moderate and heavy grazing, respectively. CSLGB was significantly reduced by 23.07% ±0.0217 , 49.73% ±0.0271 , and 97.45% ±0.0644 in the three grazing intensities. LCS significantly decreased by 21.55% ±0.0121 , 50.55% ±0.0137 , and 66.95% ±0.0449 in the three grazing intensities. RCS significantly increased by 39.23% ±0.1039 in light grazing and significantly decreased by 87.74% ±0.092 in heavy grazing. SWI significantly decreased by 21.67% ±0.0948 in moderate grazing, and SI significantly decreased by 9.67% ±0.0445 in moderate grazing. PI decreased significantly by 16.09% ±0.0785 in moderate grazing, and SR decreased significantly by 38.51% ±0.0989 and 19% ±0.081 in moderate and heavy grazing, respectively, and CI decreased significantly by 13.68% ±0.0295 in heavy grazing.

### 3.2 Effects of grazing intensity on soil and community characteristics of five primary grassland types in Xinjiang

#### 3.2.1 Soil and community characteristics of temperate meadow steppe

**Figure 3:**
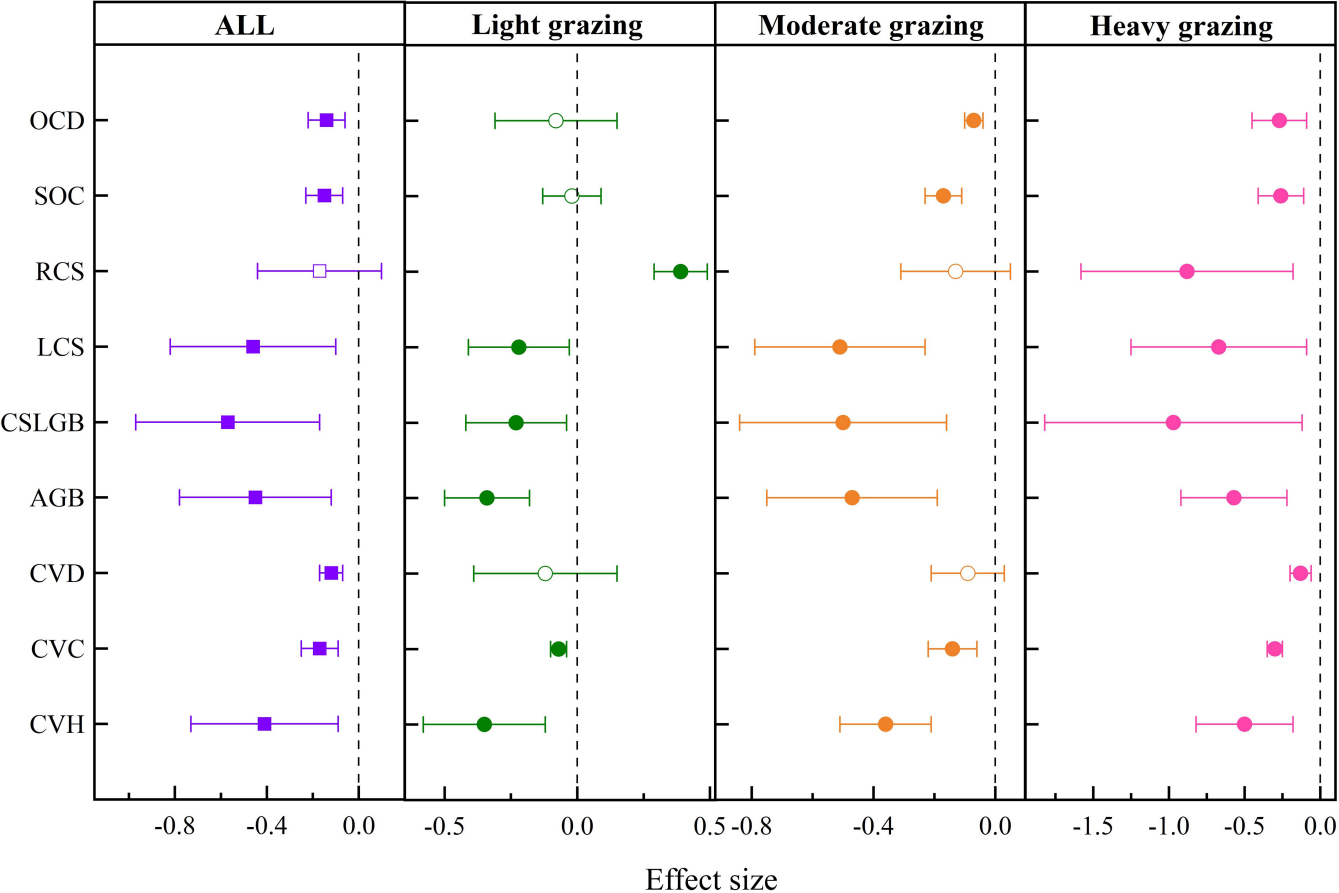
Response of soil and community characteristics to grazing intensity in temperate meadow steppe. OCD, organic carbon density; SOC, soil organic carbon; RCS, root carbon storage; LCS, litter carbon storage; CSLGB, carbon storage in live grassland biomass; AGB, aboveground biomass; CVD, community vegetation density; CVC, community vegetation coverage; CVH, community vegetation height. The solid representation has a significant effect, and the hollow representation has no significant effect.

Grazing had significant negative effects on SOC (−15.29% ±0.0325 ), OCD (−14.4% ±0.0312 ), CVH (−41.19% ± 0.0458), CVC (−16.87% ± 0.045), CVD (−11.6% ± 0.0367), AGB (−45.35% ± 0.0597), CSLGB (−56.68% ± 0.0849), LCS (−46.29% ± 0.0524), and RCS (−17.38% ± 0.1776) in temperate meadow steppe (**Figure 3**).

As shown in **Figure 3**, compared with no grazing, SOC decreased by 17.32% ±0.0558 and 25.76% ±0.0568 , and OCD decreased by 6.52% ±0.0188 and 27.2% ±0.0464 in moderate and heavy grazing, respectively. CVH decreased significantly by 35.29% ±0.063 , 36.17% ±0.0568 , and 50.14% ±0.0905 in the three grazing intensities. CVC decreased significantly by 6.94% ±0.0214 , 14.4% ±0.0306 , and 29.95% ±0.1277 in three grazing intensities. CVD decreased significantly by 12.54% ±0.0264 in heavy grazing, and AGB decreased significantly by 34.09% ±0.0902 , 46.64% ±0.0953 , and 57.17% ±0.1151 in the three grazing intensities. CSLGB decreased significantly by 23.07% ±0.0217 , 49.73% ±0.0271 , and 97.45% ±0.0644 in the three grazing intensities. LCS decreased significantly by 21.55% ±0.0121 , 50.55% ±0.0137 , and 66.95% ±0.0449 in the three grazing intensities. RCS increased significantly by 39.23% ±0.1039 in light grazing and decreased significantly by 87.74% ±0.092 in heavy grazing.

#### 3.2.2 Soil and community characteristics of temperate steppe

**Figure 4:**
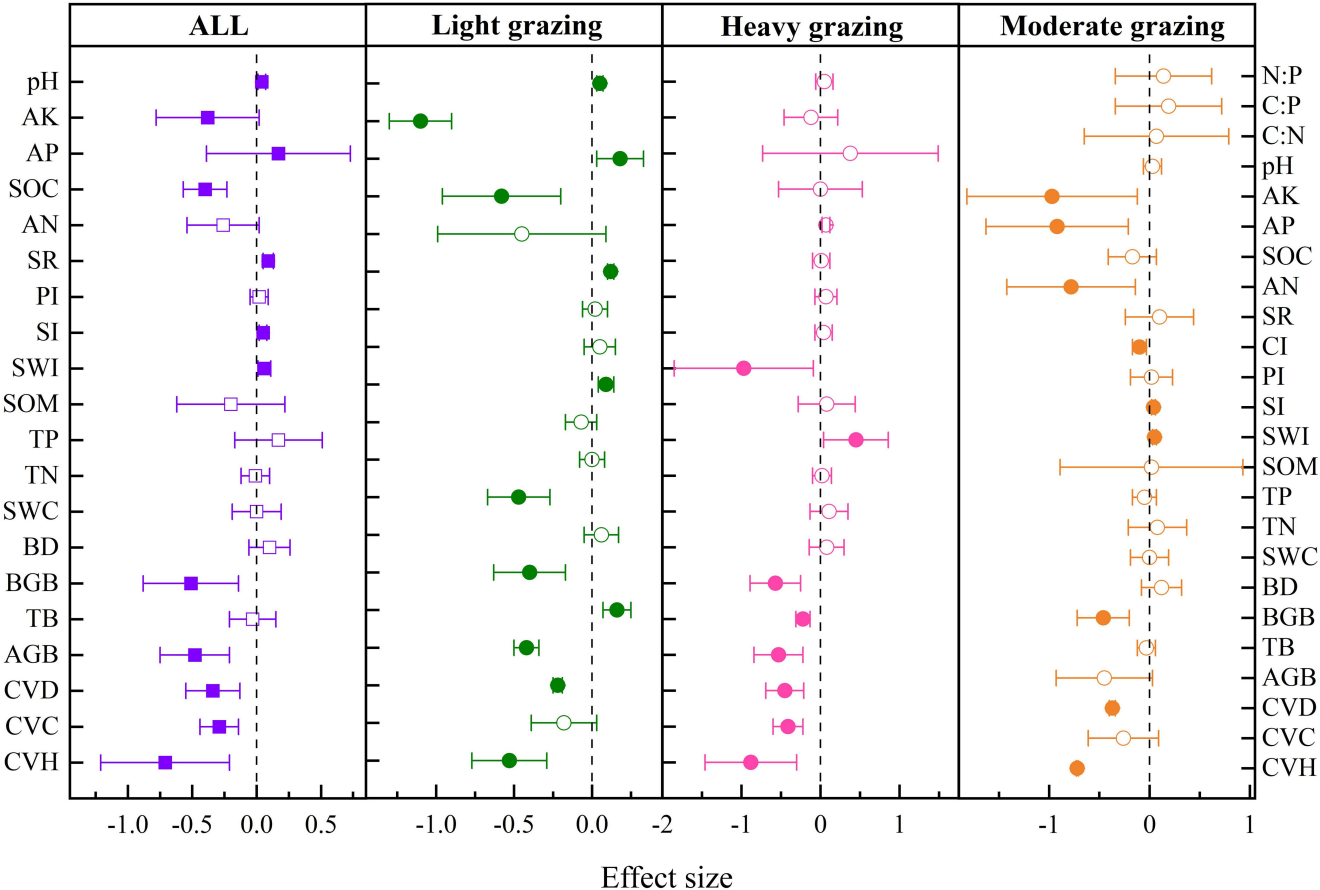
Effects of grazing intensity on soil and community indices in temperate steppe. N:P, nitrogen-to-phosphorus ratio; C:P, carbon-to-phosphorus ratio; C:N, carbon-to-nitrogen ratio; pH, soil pH; AK, available potassium; AP, available phosphorus; SOC, soil organic carbon; AN, available nitrogen; SR, species richness; CI, Chao index; PI, Pielous index; SI, Simpson index; SWI, Shannon-Wiener index; SOM, soil organic matter; TP, total phosphorus; TN, total nitrogen; SWC, soil water content; BD, bulk density; BGB, belowground biomass; TB, total biomass; AGB, aboveground biomass; CVD, community vegetation density; CVC, community vegetation coverage; CVH, community vegetation height. The solid representation has a significant effect, and the hollow representation has no significant effect.

Grazing had significant negative effects on SOC (−40.22% ±0.1201 ), CVH (−1.05% ±0.1093 ), CVC (−29.48% ±0.0722 ), CVD (−33.92% ±0.0688 ), AGB (−47.61% ±0.1036 ), BGB (−50.81% ±0.0728 ), and CI (−9.73% ±0.0124 ). There were significant positive effects on soil bulk density (BD) (9.65% ±0.0328 ), pH (4.02% ±0.0197 ), SWI (5.8% ±0.0181 ), SI (4.95% ±0.0162 ), and SR (9.36% ±0.025 ) (**Figure 4**).

As shown in **Figure 4**, compared with no grazing, soil TN decreased significantly by 47.19% ±0.14 in light grazing, soil TP increased significantly by 44.95% ±0.132 in heavy grazing, SOC decreased significantly by 57.73% ±0.1008 in light grazing, SOM decreased significantly by 96.66% ±0.0421 in heavy grazing, and soil BD increased significantly by 12.25% ±0.0391 in heavy grazing. AN decreased significantly by 78.29% ±0.0745 in moderate grazing. AP increased significantly by 17.99% ±0.0859 in light grazing and decreased significantly by 92.15% ±0.1058 in moderate grazing. AK decreased significantly by 109.52% ±0.0542 and 96.94% ±0.0633 in light and moderate grazing, respectively. Soil pH significantly increased by 4.8% ±0.0113 in light grazing, and CI was significantly decreased by 9.73% ±0.0124 in moderate grazing. CVH decreased significantly by 52.72% ±0.147 , 71.82% ±0.3599 , and 88.09% ±0.1539 in the three grazing intensities. CVC decreased significantly by 40.86% ±0.11 in heavy grazing. CVD decreased significantly by 22.01% ±0.0956 , 37.22% ±0.1769 , and 45.48% ±0.1081 in the three grazing intensities. AGB decreased significantly by 42.36% ±0.173 and 52.94% ±0.1145 in light and heavy grazing, respectively. BGB decreased significantly by 39.87% ±0.0882 , 46.29% ±0.1047 , and 56.91% ±0.1276 in the three grazing intensities. TB increased significantly by 16.32% ±0.0632 in light grazing and significantly decreased by 21.6% ±0.0632 in heavy grazing. SWI increased significantly by 8.68% ±0.0344 and 4.66% ±0.0193 in light and moderate grazing, respectively. SI increased significantly by 3.55% ±0.0169 in moderate grazing, and SR increased significantly by 12.44% ±0.1244 and 7.11% ±0.0203 in light and heavy grazing, respectively.

#### 3.2.3 Soil and community characteristics of the temperate desert steppe

**Figure 5:**
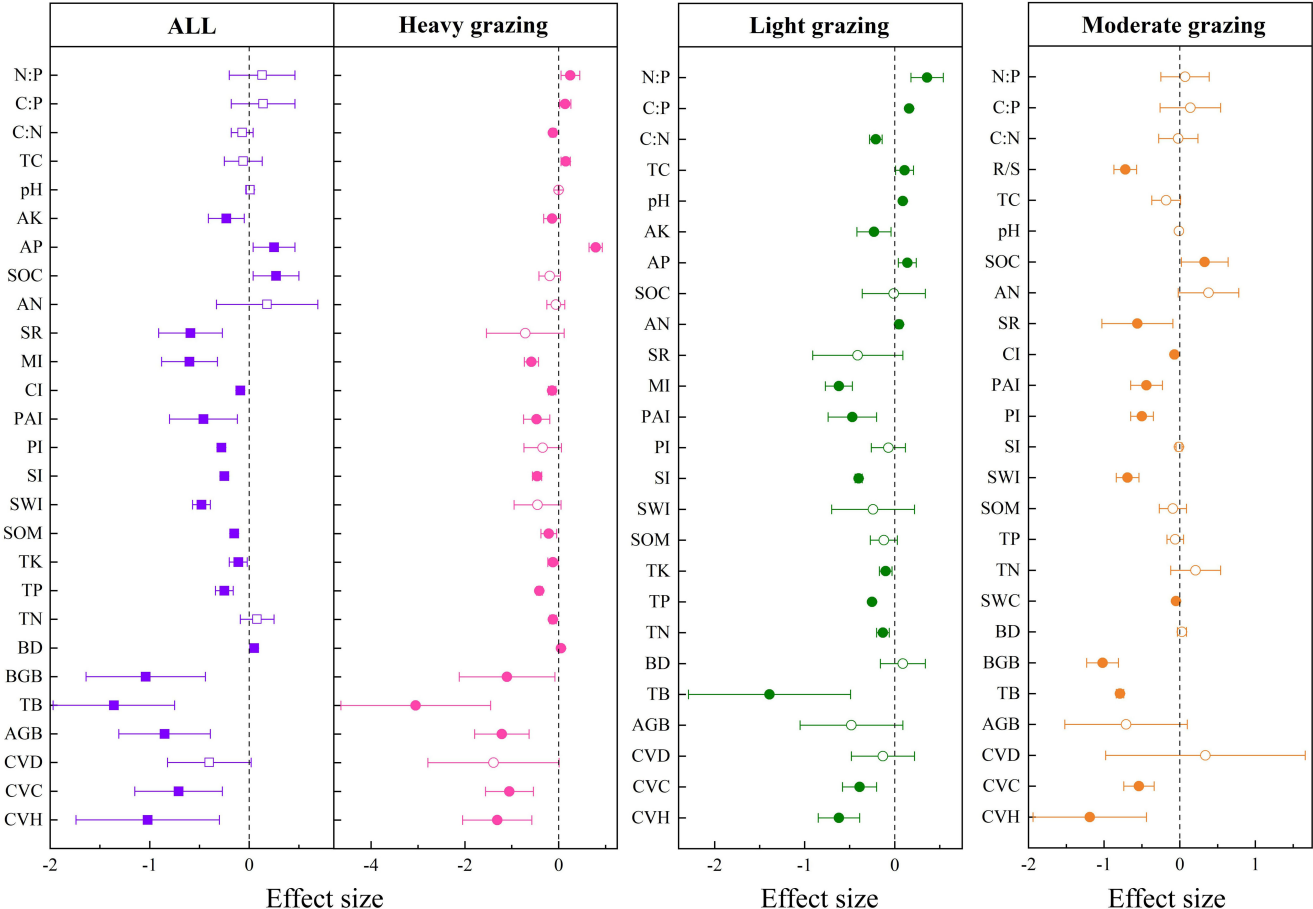
Response of soil and community indices to grazing intensity in temperate desert steppe. N:P, nitrogen-to-phosphorus ratio; C:P, carbon-to-phosphorus ratio; C:N, carbon-to-nitrogen ratio; R/S, root-to-stem ratio; TC, total carbon; pH, soil pH; AK, available potassium; AP, available phosphorus; SOC, soil organic carbon; AN, available nitrogen; SR, species richness; MI, Margalef index; CI, Chao index; PAI, Patrick index; PI, Pielous index; SI, Simpson index; SWI, Shannon-Wiener index; SOM, soil organic matter; TK, total potassium; TP, total phosphorus; TN, total nitrogen; SWC, soil water content; BD, bulk density; BGB, belowground biomass; TB, total biomass; AGB, aboveground biomass; CVD, community vegetation density; CVC, community vegetation coverage; CVH, community vegetation height. The solid representation has a significant effect, and the hollow representation has no significant effect.

Grazing had a significant negative effect on SWC (−5.38% ±0.0157 ), SOM (−14.88% ±0.0571 ), soil TP (−24.66% ±0.0803 ), soil TK (−11.13% ±0.0099 ), AK (−22.88% ±0.0227 ), CVH (−101.81% ±0.1549 ), CVC (−71.01% ±0.1384 ), AGB (−85.21% ±0.1985 ), TB (−136.06% ±0.3814 ), BGB (−104.04% ±0.2261 ), R:S (−72.45% ±0.2927 ), SWI (−47.78% ±0.2016 ), SI (−25.11% ±0.1107 ), PI (−28.45% ±0.1243 ), SR (−59.07% ±0.1364 ), CI (−8.97% ±0.0242 ), PAI (−46.4% ±0.0618 ), and MI (−59.72% ±0.1603 ). It had a significant positive effect on soil BD (5.01% ±0.0235 ), SOC (26.97% ±0.082 ), and AP (25.37% ±0.0812 ) (**Figure 5**).

As shown in **Figure 5**, compared with no grazing, soil BD increased significantly by 4.85% ±0.0077 in heavy grazing, and SWC decreased significantly by 5.38% ±0.0157 in moderate grazing. Soil pH increased significantly by 8.99% ±0.0165 in light grazing and decreased significantly by 0.97% ±0.0049 in moderate grazing. Soil TC increased significantly by 10.82% ±0.0297 in light grazing and 14.71% ±0.03 in heavy grazing. SOC increased significantly by 32.98% ±0.0897 in moderate grazing, and SOM decreased significantly by 21.11% ±0.0212 in heavy grazing. Soil TN decreased significantly by 13.11% ±0.0313 and 12.16% ±0.0286 in light and heavy grazing, respectively, and increased significantly by 21.38% ±0.0618 in moderate grazing. Soil TP decreased significantly by 24.56% ±0.1217 and 41.5% ±0.1792 in light and heavy grazing, respectively. Soil TK decreased significantly by 10.16% ±0.017 and 11.57% ±0.0073 in light and heavy grazing, respectively. AN increased significantly by 5.27% ±0.021 in light grazing. AP increased significantly by 14% ±0.0176 and 79.29% ±0.1776 in light and heavy grazing, respectively. AK decreased significantly by 23.05% ±0.0229 in light grazing. C:N decreased significantly by 21.18% ±0.0735 and 11.91% ±0.0318 in light and heavy grazing, respectively. C:P increased significantly by 16.3% ±0.0823 and 14.24% ±0.0697 in light and heavy grazing, respectively. N:P increased significantly by 36.18% ±0.0892 and 25.26% ±0.0977 in light and heavy grazing, respectively. CVH decreased significantly by 61.69% ±0.1984 , 119.13% ±0.2247 , and 130.99% ±0.291 in the three grazing intensities. CVC decreased significantly by 39.2% ±0.1026 ,54.38% ±0.1766 , and 105.34% ±0.2782 in three grazing intensities. AGB decreased significantly by 121.33% ±0.3224 in heavy grazing. TB decreased significantly by 138.52% ±0.2462 , 78.95% ±0.375 , and 305.1% ±0.7415 in the three grazing intensities. BGB decreased significantly by 101.57% ±0.4143 and 109.91% ±0.042 in moderate and heavy grazing, respectively. R:S was significantly decreased by 72.45% ±0.2927 in moderate grazing. SWI significantly decreased by 68.85% ±0.2728 in moderate grazing. SI significantly decreased by 40% ±0.1837 and 45.76% ±0.18 in light and heavy grazing, respectively. PI significantly decreased by 50.25% ±0.1812 in moderate grazing. SR decreased significantly by 55.51% ±0.0421 in moderate grazing. PAI decreased significantly by 47.34% ±0.1059 , 44.06% ±0.1154 , and 47.34% ±0.1011 in the three grazing intensities. CI decreased significantly by 6.54% ±0.0189 and 13.68% ±0.0295 in moderate and heavy grazing, respectively. MI decreased significantly by 61.9% ±0.238 and 57.9% ±0.217 in light and heavy grazing, respectively.

#### 3.2.4 Soil and community characteristics of mountain meadow

**Figure 6:**
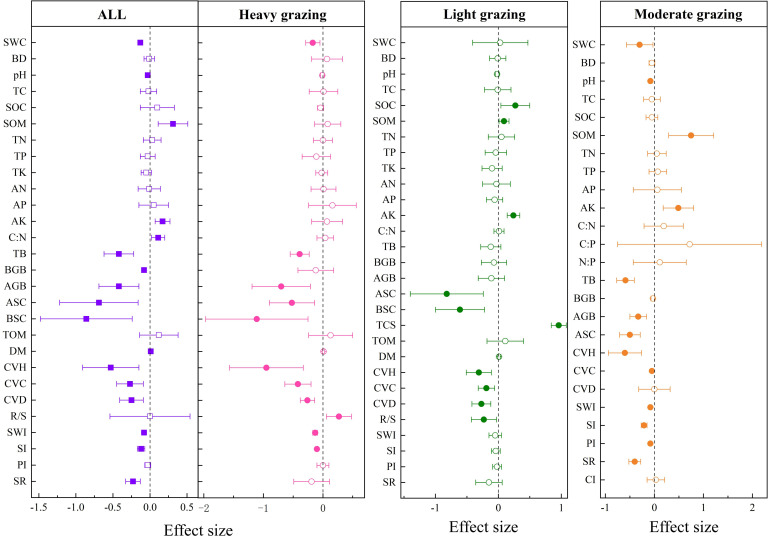
Effects of grazing intensity on soil and community properties in mountain meadow. SWC, soil water content; BD, bulk density; pH, soil pH; TC, total carbon; SOC, soil organic carbon; SOM, soil organic matter; TN, total nitrogen; TP, total phosphorus; TK, total potassium; AN, available nitrogen; AP, available phosphorus; AK, available potassium; C:N, carbon-to-citrogen ratio; C:P, carbon-to-phosphorus ratio; N:P, nitrogen-to-phosphorus ratio; TB, total biomass; BGB, belowground biomass; AGB, aboveground biomass; ASC, aboveground standing crop; BSC, belowground standing crop; TCS, total carbon storage; TOM, total organic matter; DM, dry matter; CVH, community vegetation height; CVC, community vegetation coverage; CVD, community vegetation density; R:S, root-to-stem ratio; SWI, Shannon-Wiener index; SI, Simpson index; PI, Pielous index; SR, species richness; CI, Chao index. The solid representation has a significant effect, and the hollow representation has no significant effect.

Grazing had significant negative effects on SWC (−12.77% ±0.0569 ), pH (−2.64% ±0.0123 ), CVH (−53.08% ±0.0766 ), CVC (−27.02% ±0.0436 ), CVD (−25.51% ±0.0471 ), AGB (−42.19% ±0.0762 ), TB (−42.06% ±0.1102 ), BGB (−8.18% ±0.0362 ), ASC (−69.11% ±0.0799 ), BSC (85.66% ±0.1224 ), SWI (−8.32% ±0.0283 ), SI (−12.25% ±0.0376 ), and SR (−23.04% ±0.0659 ). We identified a significant positive effect on SOM (30.88% ±0.1468 ), AK (17.29% ±0.0664 ), C:N (10.56% ±0.051 ), DM (0.61% ±0.002 ), and TCS (95.76% ±0.0809 ) (**Figure 6**).

As shown in **Figure 6**, compared with no grazing, SWC decreased significantly by 30.23% ±0.0171 and 17.13% ±0.0263 in moderate and heavy grazing, respectively. Soil pH decreased significantly by 7.5% ±0.0274 in moderate grazing, and SOC increased significantly by 27.48% ±0.1316 in light grazing. SOM increased significantly by 9.44% ±0.0434 and 75.18% ±0.3609 in light and moderate grazing, respectively. AK increased significantly by 24.27% ±0.0865 and 48.76% ±0.1624 in light and moderate grazing, respectively. CVH decreased significantly by 30.62% ±0.0567 , 59.67% ±0.1327 , and 94.56% ±0.1648 in the three grazing intensities. CVC decreased significantly by 18.56% ±0.0286 , 5.22% ±0.0326 , and 41.56% ±0.1005 in the three grazing intensities. CVD decreased significantly by 27.46% ±0.0631 and 25.57% ±0.0691 in light and heavy grazing, respectively. AGB decreased significantly by 32.66% ±0.0811 and 70.01% ±0.1046 in moderate and heavy grazing, respectively. TB decreased significantly by 59.17% ±0.2081 and 39.19% ±0.1206 in moderate and heavy grazing, respectively. ASC was significantly reduced by 82.04% ±0.123 , 50.48% ±0.148 , and 52.2% ±0.0721 in the three grazing intensities. BSC was significantly decreased by 60.61% ±0.1125 and 110.55% ±0.1276 in light and heavy grazing, respectively. R:S was significantly decreased by 27.84% ±0.0177 in light grazing and increased by 27.3% ±0.0175 in heavy grazing. DM significantly increased by 0.75% ±0.0022 in heavy grazing. TCS significantly increased by 95.76% ±0.0809 in light grazing. SWI was significantly decreased by 7.72% ±0.0241 and 13.15% ±0.0468 in moderate and heavy grazing, respectively. SI significantly decreased by 20.61% ±0.074 and 9.56% ±0.0367 in moderate and heavy grazing, respectively. PI decreased by 8.46% ±0.041 in moderate grazing, and SR decreased by 40.18% ±0.1443 in moderate grazing.

#### 3.2.5 Soil and community characteristics of alpine meadow

**Figure 7:**
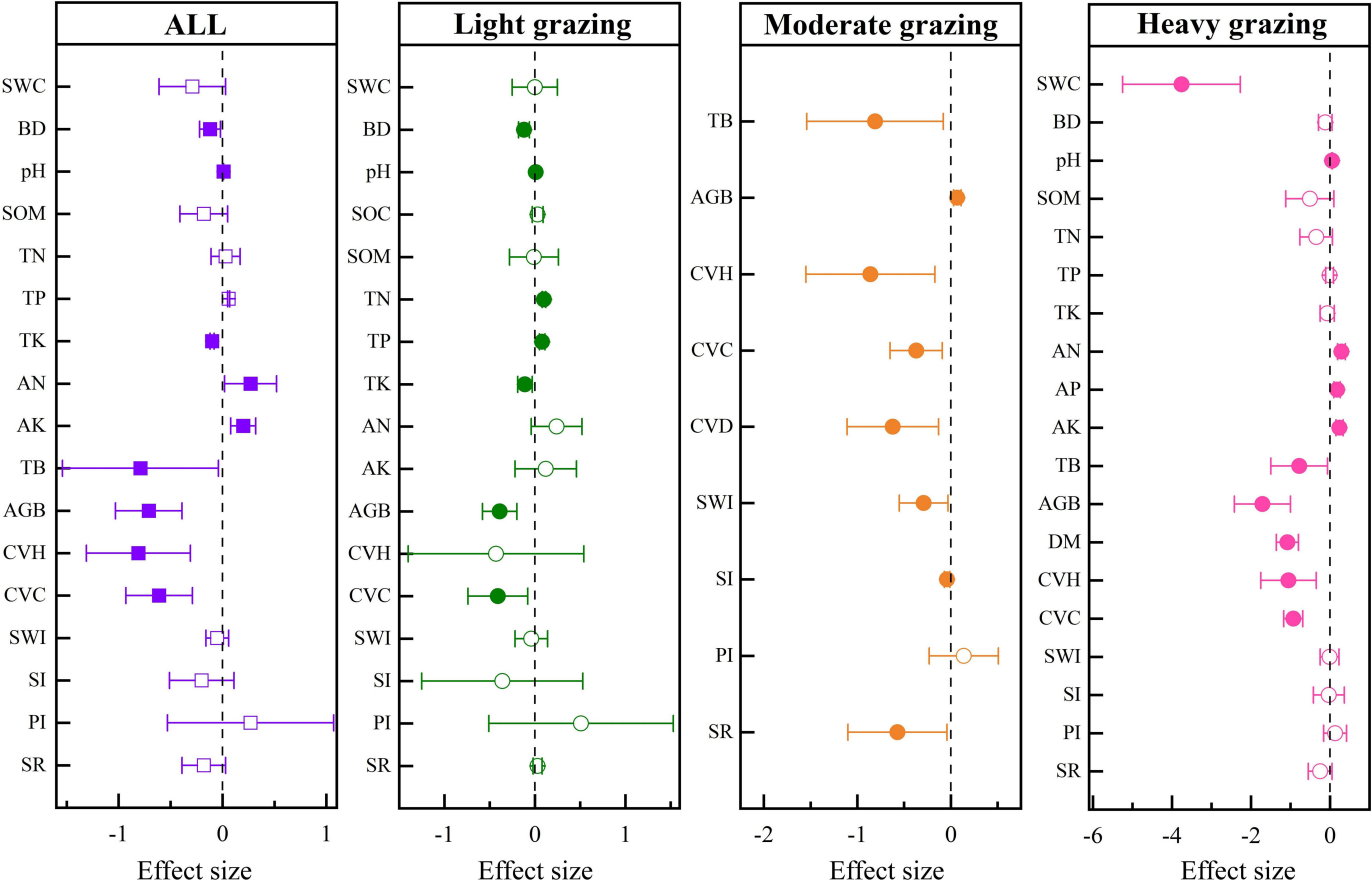
Response of soil and community characteristics to grazing intensity in alpine meadow. SWC, soil water content; BD, bulk density; pH, soil pH; SOC, soil organic carbon; SOM, soil organic matter; TN, total nitrogen; TP, total phosphorus; TK, total potassium; AN, available nitrogen; AP, available phosphorus; AK, available potassium; TB, total biomass; AGB, aboveground biomass; DM, dry matter; CVH, community vegetation height; CVC, community vegetation coverage; CVD, community vegetation density; SWI, Shannon-Wiener index; SI, Simpson index; PI, Pielous index; SR, species richness. The solid representation has a significant effect, and the hollow representation has no significant effect.

Grazing had significant negative effects on soil BD (−12.33% ±0.0125 ), TK (−9.58% ±0.0415 ), CVH(−80.93% ±0.1582 ), CVC (−61.39% ±0.1517 ), CVD (−62.31% ±0.0642 ), AGB (−71.21% ±0.2015 ), TB (−79.08% ±0.0235 ), and DM (−107.52% ±0.4036 ). Grazing had significant positive effects on soil pH (1.12% ±0.0042 ), TP (6.02% ±0.0254 ), AN(26.68% ±0.0919 ), AP (18.49% ±0.0506 ), and AK (19.72% ±0.0613 ) (**Figure 7**).

As shown in **Figure 7**, compared with no grazing, SWC decreased significantly by 375.61% ±0.1356 in heavy grazing, and soil BD decreased significantly by 12.07% ±0.0204 in light grazing. Soil TN increased significantly by 9.47% ±0.0382 in light grazing, TP increased significantly by 8.38% ±0.0274 in light grazing, TK decreased significantly by 10.64% ±0.0374 in light grazing, and pH increased significantly by 0.96% ±0.0043 and 5.12% ±0.0194 in light and heavy grazing. AN increased significantly by 28.56% ±0.0926 in heavy grazing, AP increased significantly by 18.49% ±0.0506 in heavy grazing, and AK increased significantly by 23.67% ±0.0743 in heavy grazing. CVH decreased significantly by 86.12% ±0.086 and 105.13% ±0.1791 in moderate and heavy grazing, respectively. CVC decreased significantly by 41.3% ±0.041 , 36.89% ±0.0455 , and 92.94% ±0.3537 in the three grazing intensities. CVD decreased significantly by 62.31% ±0.0642 in moderate grazing. AGB decreased significantly by 39.28% ±0.1018 and 171.03% ±0.5111 in light and heavy grazing, respectively, and increased significantly by 7.42% ±0.0354 in moderate grazing. TB decreased significantly by 81.16% ±0.0414 and 78% ±0.078 in moderate and heavy grazing, respectively. SWI decreased significantly by 29.33% ±0.0192 in moderate grazing, SI decreased significantly by 4.17% ±0.0059 in moderate grazing, and SR decreased significantly by 56.6% ±0.0181 in moderate grazing. DM decreased significantly by 107.52% ±0.4036 in heavy grazing.

### 3.3 Effects of grazing intensity on different grassland types

By integrating the similarities and differences of the effects of different grazing intensities on various grassland types (**Table 2**), grazing reduced the vegetation community height of various grassland types as a whole. In addition, and heavy grazing reduced the vegetation community height, coverage, and aboveground and underground biomass of various grassland types. Light grazing decreased the SOC of temperate steppe and increased the SOC of temperate desert steppe. Moderate grazing reduced the AGB of mountain meadow and increased the AGB of alpine meadow. Moderate grazing decreased SOC in temperate meadow steppe and increased SOC in temperate desert steppe.

**Table 2:** Similarities and differences of different grassland types in response to grazing.

Grassland type	Differences			Similarities
	LG	MG	HG	
Temperate meadow steppe		SOC↓17.32%*	SOC↓25.8%*	The SWC (except for temperate meadow steppe) of all grassland types decreased significantly. TK was continuously lost and AP was enriched. All types of CVH were significantly reduced. CVH, CVC and AGB decreased significantly under heavy grazing. SR of each grassland type (except for the significant increase of 12.4 % in temperate steppe) decreased significantly under moderate grazing. SWI of each grassland type (except for the significant increase of 5.8 % in temperate meadow steppe) decreased significantly.
Temperate steppe	SOC↓57.7%* pH↑4.8%* SR↑12.4% * TN↓47.19%*	AP↓92.15%* SWI↑4.66%*	CVC↓40.86%*	
Temperate desert steppe	SOC↑32.98%*	SOC↑33.0%*	CVC↓105.34%* AP↑79.3%*	
Mountain meadow	TCS↑95.76%*	AGB↓70.01%*	SWC↓17.13%*	
Alpine meadow	AGB↑7.42%* TN↑9.47%*	AGB↑7.4%*	CVC↓92.94%*	

*, * *, and * * * indicated significance (*P*) less than 0.05,0.01, and 0.001; LG, light grazing; MG, moderate grazing; HG, heavy grazing; SWC, soil water content; SOC, soil organic carbon; TN, total nitrogen; TK, total potassium; AP, available phosphorus; AGB, aboveground biomass; TCS, total carbon storage; CVH, community vegetation height; CVC, community vegetation coverage; CVD, community vegetation density; SWI, Shannon-Wiener index; SR, species richness;

The interaction effects of grassland type and grazing intensity on all key indicators were significant (*P* < 0.05; **Table 3**), confirming that the impact of grazing had a strong ecosystem dependence. The temperate meadow steppe was highly sensitive to the loss of soil organic carbon (SOC: β = −0.42, P = 0.01) and aboveground biomass (AGB: β = −1.2, P < 0.001). The temperate steppe showed significant changes in soil physical properties, including increased pH under light grazing (β = 0.3, P = 0.03) and increased bulk density under heavy grazing (β = 0.15, P = 0.04). The temperate desert steppe was the only ecosystem with a significant increase in SOC under moderate grazing (β = 0.28, P = 0.02), but its biodiversity (SI: β = −0.8, P = 0.001) was severely damaged. AK in mountain meadows was significantly accumulated under light and moderate grazing (β = 0.35, P = 0.01), while heavy grazing induced the allocation of resources to the underground (R/S: β = 0.25, P = 0.03). The alpine meadow showed a contradictory response: although moderate grazing increased aboveground biomass (AGB: β = 0.4, P = 0.04), it decreased total biomass (TB: β = −0.6, P = 0.02), which suggested a transformation of community structure.

**Table 3:** Different responses of different grassland types to grazing.

Grassland type	Unique response characteristics	Interaction test (Grassland type × Grazing intensity)
Temperate meadow steppe	Moderate and heavy grazing reduced SOC content (*β* = -0.42, *P* = 0.01) Different grazing intensities reduced AGB (*β* =-1.2, *P* <0.001)	*P* =0.002
Temperate steppe	Light grazing increased pH (*β* = 0.3, *P* = 0.03) Heavy grazing increased soil BD (*β* = 0.15, *P* = 0.04)	*P* =0.008
Temperate desert steppe	Moderate grazing increased SOC (*β* = 0.28, *P* = 0.02) Moderate grazing reduced SI (*β* = -0.8, *P* = 0.001)	*P* =0.013
Mountain meadow	Light and moderate grazing increased AK (*β* = 0.35, *P* = 0.01) Heavy grazing increased R/S (*β* = 0.25, *P* = 0.03)	*P* =0.004
Alpine meadow	Moderate grazing reduced TB (*β* = -0.6, *P* = 0.02) Moderate grazing increased AGB (*β* = 0.4, *P* = 0.04)	*P* =0.006

### 3.4 The relationship between community vegetation characteristics and the diversity index of grassland

**Figure 8:**
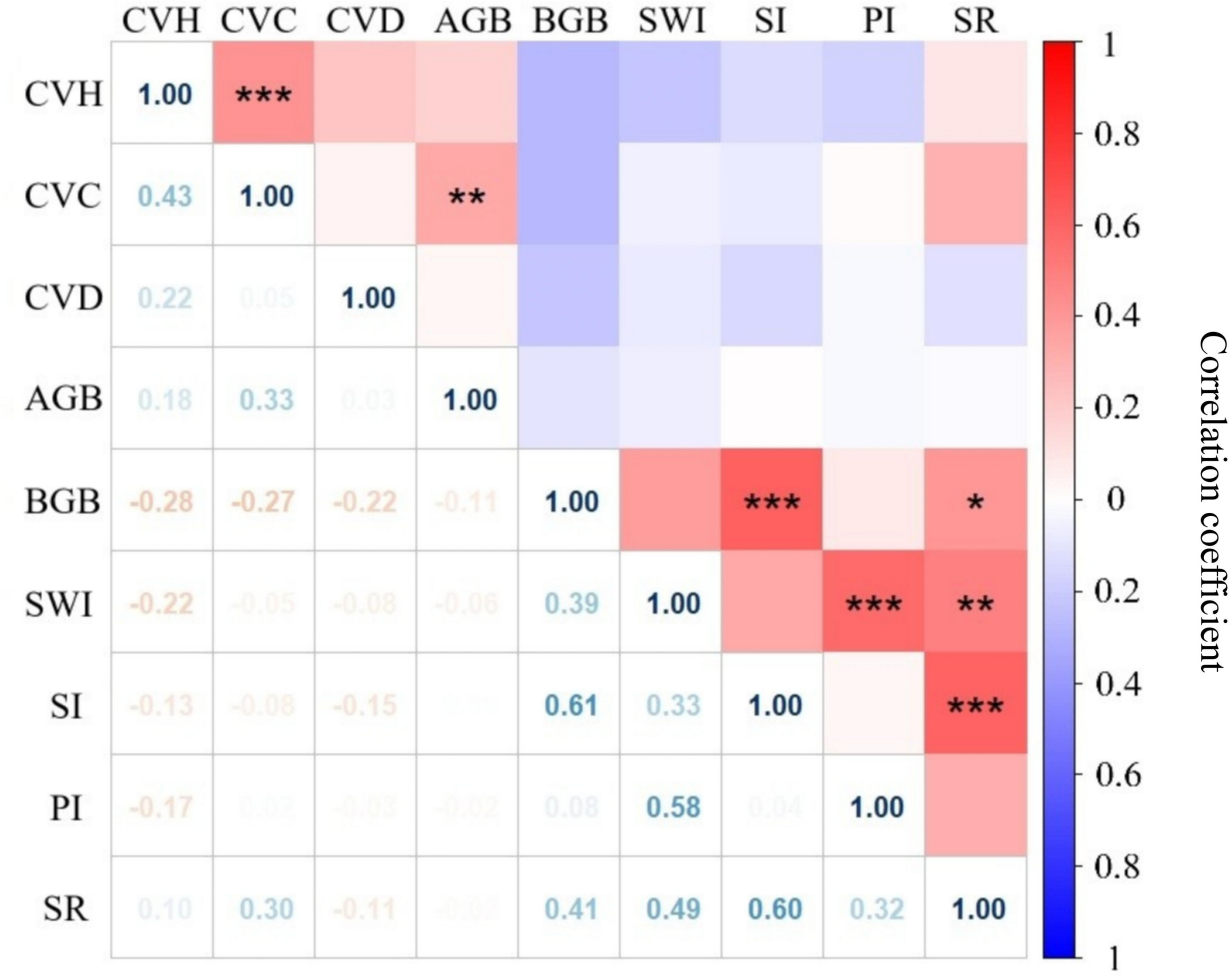
Correlation of each index of grassland community vegetation characteristics in Xinjiang. *, * *, and * * * indicated significance (*P*) less than 0.05,0.01, and 0.001, respectively. CVH, community vegetation height; CVC, community vegetation coverage; CVD, community vegetation density; AGB, aboveground biomass; BGB, belowground biomass; SWI, Shannon-Wiener index; SI, Simpson index; PI, Pielous index; SR, species richness.

According to the correlation analysis of vegetation characteristics and diversity index, a significant positive correlation existed between CVH and CVC (*P* < 0.001), and there was a significant positive correlation existed between CVC and AGB (*P* < 0.01). BGB was significantly positively correlated with SI (*P* < 0.001) and SR (*P* < 0.05). SWI was significantly positively correlated with PI (*P* < 0.001) and SR (*P* < 0.01). SI was positively correlated with SR (*P* < 0.001) (**Figure 8**). These results demonstrated that an increase in grassland vegetation height promoted the expansion of coverage and then an increase in AGB. The increase in BGB promoted the increase in SR and diversity index.

### 3.5 The influence path of grazing intensity on grassland species diversity and soil quality in Xinjiang

**Figure 9:**
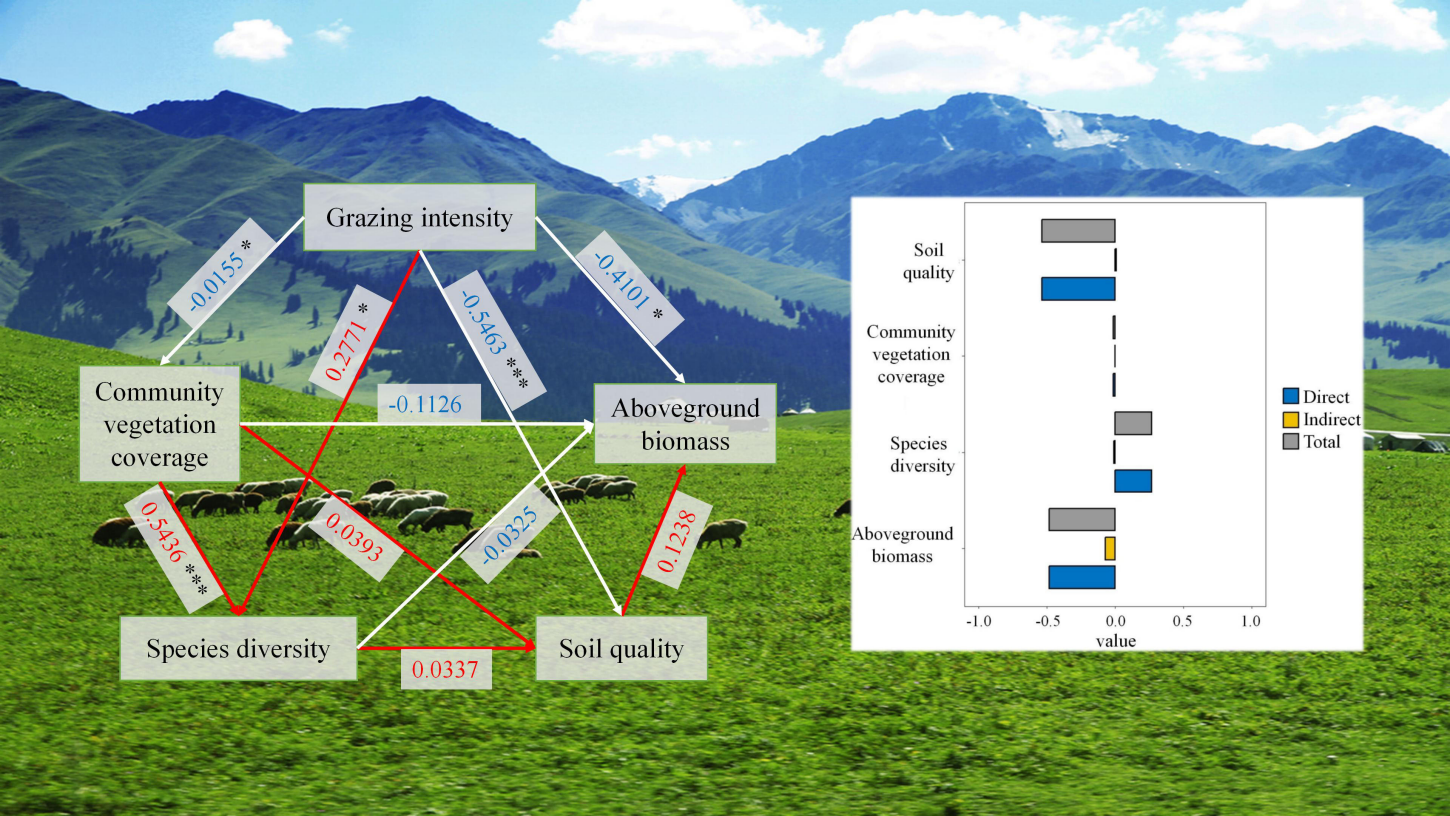
Effects of grazing intensity on grassland species diversity and soil quality in Xinjiang. “*” and “***” are the significant codes of P (significance) < 0.05 and P (significance) < 0.001 for the mean effect size, respectively.

The influence path of different grazing intensities on soil quality and species diversity in Xinjiang grassland is shown in **Figure 9**. Grazing intensity is significantly negatively correlated with CVC (*P* < 0.05), and CVC is significantly positively correlated with species diversity (*P* < 0.001). Therefore, grazing intensity affected species diversity by affecting vegetation coverage. The greater the grazing intensity, the smaller the vegetation coverage, which resulted in a decrease in species diversity. In addition, grazing intensity was significantly negatively correlated with AGB (*P* < 0.05) and soil quality (*P* < 0.001). Increasing grazing intensity led to a decrease in AGB and a decrease in soil quality.

## 4 Discussion

### 4.1 Effects of different grazing intensities on soil physical and chemical properties of grassland

Grazing intensity significantly affected the physical and chemical properties of the soil. With the increase in grazing intensity, the BD of the soil surface (0–10cm) usually increased, and the soil became compact. The results of this study showed that light and moderate grazing reduced soil BD in Xinjiang grassland, whereas heavy grazing increased soil BD, which was similar to the results of Zhang et al. (2002). SWC decreased significantly under moderate and heavy grazing conditions. It is possible that livestock frequently trampled the grassland, compacted the soil, reduced the porosity, reduced the storage space of air and water in the soil, and hindered the infiltration and retention of water, thus leading to a decrease in SWC.

The soil pH of the temperate desert steppe in Xinjiang increased and decreased significantly under light and moderate grazing, respectively. The reason may be that light grazing reduced CVC and accelerated soil moisture evaporation. The soluble salts in the soil may have gradually accumulated on the surface, thus increasing soil pH. Moderate grazing accelerated the decomposition of SOM and released more organic acids. These organic acids would neutralize the alkaline substances in the soil, thereby reducing the soil pH (An and Xu, 2013). SOC, TC, and TN decreased significantly under heavy grazing. SOC decreased under light and moderate grazing conditions in two types of grassland in Xinjiang temperate meadow steppe and temperate steppe, but the SOC content in temperate desert steppe and mountain meadow increased significantly under light and moderate grazing. This may have been the result of a decrease in CVC under light and moderate grazing conditions in temperate meadow steppe and temperate steppe, which resulted in a decrease in plant residue input, a corresponding decrease in SOC accumulation, a destruction of soil structure, and a decrease in porosity. This, in turn, may have affected the soil microbial activity and organic carbon fixation (Ding et al., 2014). The vegetation types of temperate desert steppe and mountain meadow usually have strong adaptability and can better cope with grazing pressure. Li et al. (2008) have shown that plants such as *Stipa breviflora* in desert steppe can maintain a good growth state under light and moderate grazing, and their root exudates and litter input contributed to the accumulation of SOC. TP in alpine meadow soil increased significantly under light grazing, which was similar to the results of Wu et al. (2024b). It is possible that grazing changed the growth strategy of plants, further increasing plant root exudates. These exudates promoted the dissolution and release of phosphorus in the soil, which increased the TP content in the soil.

### 4.2 Effects of different grazing intensities on grassland community structure characteristics

Studies have shown that grazing is an important factor in the dominant community structure when the climatic conditions are basically the same (He et al., 2021). The results of this study showed that the CVH, CVC, and CVD in various grassland types in Xinjiang decreased significantly under different grazing intensities, which was consistent with the research results of Zhang et al. (2021) on mountain meadow in Xinjiang that is, CVD, CVH, and CVC under grazing conditions were lower than those in enclosed areas. The study of *S. purpurea* grassland in northern Tibet by Duan et al. (2010) also found that high-intensity grazing behavior significantly reduced CVC, which indicated that the trampling and grazing of livestock under grazing conditions affected the growth of CVD, CVH, and CVC. The results of this study also showed that grazing reduced the AGB, BGB, and TB of different grassland types in Xinjiang, which was consistent with the results of previous studies (Wang et al., 2009; Yu et al., 2015). We determined that grazing reduced the number of plants and the total photosynthetic area of plants, thus resulting in a decrease in the efficiency of organic matter accumulation. Compared with no grazing, the R:S of heavy grazing was greater, which indicated indicating that grazing gradually reduced the that TB of the community and the TB of the community gradually trended downward (Zhang et al., 2020b). Litter biomass also decreased with the increase in grazing intensity, because grazing accelerated the decomposition and removal of litter (Zhang et al., 2020b).

Light grazing had higher community species diversity than heavy grazing. The results of this study showed that light and moderate grazing increased SR and SWI in temperate steppe of Xinjiang, whereas moderate grazing in mountain meadow and alpine meadow decreased SR, SWI, and SI. It is possible that moderate grazing disturbance broke the single advantage of vegetation and provided growth space for grazing-tolerant or less palatable plants, thereby increasing species diversity. This occurred because intermediate disturbance not only inhibited the competitive exclusion by dominant species but also provided survival opportunities for some rare species (Xiang, 2017). Mountain and alpine meadow ecosystems are more sensitive to grazing disturbance. Moderate grazing was sufficient to have had a greater impact on vegetation community structure, which resulted in a decrease in species diversity. Mountain and alpine meadows usually have lower vegetation productivity and slower recovery ability. Therefore, even moderate grazing may have had a negative impact on species diversity (Cai et al., 2024). In the study of alpine meadow in eastern Qilian Mountains, Wang et al. (2019) found that the SR and SWI were significantly higher in light grazing than those under other grazing intensities, while moderate and heavy grazing reduced these diversity indexes.

## 5 Conclusion

In this study, we explored the comprehensive effects of light, moderate, and heavy grazing on soil and community structure characteristics of grassland in Xinjiang. We retrieved relevant literature and used the meta-analysis method to identify the effects of different grazing intensity on soil physical and chemical properties, community vegetation characteristics and species diversity of five primary grassland types in Xinjiang. The conclusions showed the following: (1) In terms of soil properties, grazing reduced soil TK and increased AP content as a whole, and heavy grazing reduced SOC and OCD. Under different types, light grazing increased soil pH and AP in the temperate meadow steppe. Heavy grazing increased soil TP and BD in temperate grassland. Moderate grazing reduced the SWC and pH of temperate desert steppe and mountain meadow. light and moderate grazing increased SOM and AK in mountain meadows. Light grazing increased soil pH, TN and TP contents, and decreased soil BD and TK in alpine meadow. (2) In terms of vegetation characteristics, CVC, CVD, and CVH decreased under different grazing intensities. AGB, BGB, and TB followed a downward trend under moderate and heavy grazing. Heavy grazing increased the R:S and DM content of mountain meadow vegetation. (3) In terms of species diversity, moderate grazing reduced SR, SWI, SI, and PI. Moderate grazing increased SWI and SI in temperate grassland of Xinjiang. Overall, results of this study show that the increase in grazing intensity reduced vegetation CVC, thus reducing species diversity.

## Data Availability

The original contributions presented in the study are included in the article/Supplementary Material. Further inquiries can be directed to the corresponding author.
